# Is It Cold Enough? Effects of Artificial and Natural Chilling on Budbreak and Frost Hardiness in *Acer saccharum* (Marsh.)

**DOI:** 10.1111/ppl.70586

**Published:** 2025-10-15

**Authors:** Claudio Mura, Guillaume Charrier, Al P. Kovaleski, Patricia Raymond, Annie Deslauriers, Sergio Rossi

**Affiliations:** ^1^ Département de Sciences fondamentales Université du Québec à Chicoutimi Chicoutimi Quebec Canada; ^2^ Université Clermont Auvergne, INRAE, UMR PIAF Clermont‐Ferrand France; ^3^ Department of Horticulture University of Wisconsin–Madison Madison Wisconsin USA; ^4^ Direction de recherche forestière Ministère des Ressources naturelles et des Forêts (MRNF) Québec Quebec Canada

**Keywords:** dormancy dynamics, endodormancy break, frost hardiness, phenology, provenances

## Abstract

A crucial part of the phenological cycle in temperate and boreal trees is the exposure to chilling temperatures releasing endodormancy, which allows the trees to react to external signals and resume growth in spring. We compared the effect of artificial and natural chilling on endodormancy break and frost hardiness of sugar maple ( *Acer saccharum* ) seedlings. Samples were either placed in growing chambers under artificial chilling conditions (4°C) or outdoors (natural temperatures, including < 0°C) in Chicoutimi, Canada. During dormancy, we performed regular transfers to forcing conditions, quantified frost hardiness (LT_50_) at the time of transfer and observed the time to budbreak (TBB). We measured chilling accumulation with classic models considering only temperatures above 0°C (Chilling Hours, Utah Model, and Dynamic Model) and with a modified model accounting for all temperatures between −10°C and 7.2°C. Samples in artificial chilling showed earlier deacclimation and initiated budbreak in late April, indicating that 4°C can both fulfill the chilling requirement and initiate ontogenetic development. Samples under natural chilling showed later deacclimation, correlating with a longer TBB. Endodormancy break point was only identified in artificial conditions, after 2715 to 3075 h at 4°C. The chilling model accounting for freezing temperatures outperformed classic chilling models. Seedling provenance did not have a significant effect. Our results indicate that including freezing temperatures can improve chilling calculations in cold climates or boreal species, where temperatures remain below 0°C during most of the winter. Moreover, measuring frost hardiness during chilling‐forcing experiments can clarify how acclimation and deacclimation influence dormancy dynamics.

## 1 Introduction

In temperate and boreal ecosystems, trees need to synchronize their annual cycle with external conditions to avoid frost damage. When freezing temperatures exceed the tree's frost hardiness, ice formation inside the cytosol can lead to cell death, tissue damage, and death (Charrier et al. 2018; Mayland and Cary 1970; Uemura et al. 2006). There is a trade‐off between growth and stress resistance, and trees improve their frost resistance by concentrating their activity in the favorable growing season and spending the winter in a dormant state (Hänninen and Tanino 2011; Volaire et al. 2023). This phenological cycle of growth and dormancy is accompanied by physiological adjustments, for example, increased frost hardiness overwinter (Charrier et al. 2015).

The main cues inducing dormancy are shortening photoperiod and decreasing temperatures in the late summer and autumn (Fuchigami et al. 1982; Hamilton et al. 2016; Rohde and Bhalerao 2007). Low temperatures can activate *Dormancy‐Associated MADS‐Box* (*DAM*) genes, which are responsible for dormancy induction (Lloret et al. 2018; Wu et al. 2017), while shortening photoperiod interacts with photoreceptors such as phytochrome A to accelerate growth cessation (Lloret et al. 2018; Olsen 2010). Different types of dormancy are identified in the literature, namely paradormancy, endodormancy, and ecodormancy (Lang et al. 1987). Paradormancy is a transition stage where growth is inhibited by factors internal to the plant but external to the bud (correlative inhibition, e.g., apical dominance), and growth resumption is still possible under favorable external conditions (Lang et al. 1987). As temperatures get colder and photoperiod shortens, trees enter the endodormancy stage. Endodormancy is characterized by endogenous inhibition of growth in the bud, which slows down loss of frost hardiness and growth resumption in response to favorable conditions, for example, late warm spells during autumn (Charrier et al. 2015; Kovaleski 2024). During endodormancy, trees increase their frost hardiness in a process called cold acclimation (Charrier et al. 2011; Sakai and Larcher 1987; Vitasse et al. 2014). Exposure to cold temperatures, that is, chilling, is necessary to break endodormancy (Chuine et al. 2016; Coville 1920), although stress such as heat waves may also induce endodormancy release (Mohamed et al. 2014). After the endodormancy break, trees enter the ecodormancy stage, which is externally regulated by environmental cues (Charrier et al. 2015; Lang et al. 1987). During ecodormancy, warm temperatures and increasing photoperiod during spring induce loss of frost hardiness (i.e., cold deacclimation), budbreak, and growth resumption (Delpierre et al. 2016; Flynn and Wolkovich 2018; Junttila 2007; Leinonen and Kramer 2002). The endodormancy break and the chilling accumulation required to break it are therefore key parts of the phenological cycle of the tree (Charrier 2022; Hänninen and Kramer 2007).

One major concern under climate change is that projected warmer winter conditions could be insufficient to fulfill chilling requirements, preventing an endodormancy break and leading to phenological maladaptation, such as delays in budbreak (Chuine et al. 2016). Assessing chilling requirements of different species and ecotypes within is therefore necessary to better predict dormancy dynamics and phenology under future conditions (Laube et al. 2014). One common approach to determine chilling requirements is through chilling‐forcing experiments, in which samples are transferred at regular intervals from chilling conditions to growing conditions (i.e., forcing) during the autumn and winter (Hänninen et al. 2019). This allows for the determination of the endodormancy break, which can be defined as the point where budbreak percentage is maximized or when budbreak stops decreasing significantly (Hänninen 2016). Chilling‐forcing experiments are sometimes conducted in controlled conditions, whereas the derived models are tested on budbreak data in natural conditions (Hänninen et al. 2019). Other studies directly perform chilling‐forcing experiments under natural conditions (e.g., Charrier et al. 2011; El Yaacoubi et al. 2016; Heide 1993).

Several models have been developed, typically in horticulture, to calculate chilling accumulation, such as the classic Chilling Hours Model (Weinberger 1950), the Utah Model (Richardson et al. 1974), and the Dynamic Model (Fishman et al. 1987). These models differ in the way that chilling unit accumulation is calculated and in the range of temperatures that are considered effective for chilling. Chilling Hours accounts for temperatures between 0°C and 7.2°C, with no contribution outside this range. The Utah Model considers temperatures between 1.4°C and 12.4°C, with different weights at set temperature ranges and a negative effect (chilling negation) at temperatures warmer than 15.9°C. The Dynamic Model is a two‐step model in which warm temperatures can negate chilling only in the first step. For chilling computation, the Dynamic Model assumes a bell‐shaped curve for chilling accumulation, between −2°C and 12.6°C, with maximum efficiency at 6°C (Erez et al. 1990). A shared feature of these models is the assumption that temperatures below 0°C have little to no effect on chilling accumulation. However, this assumption is not based on a mechanistic understanding of the physiological processes underlying chilling requirements, which is still lacking (Fadón et al. 2020; North et al. 2024; Wang et al. 2020). In colder environments where temperatures remain below 0°C for several months (i.e., cold temperate, high latitude or elevated environments), the above‐mentioned chilling models could therefore lead to underestimation of chilling accumulation. Indeed, several authors support the addition of freezing temperatures in chilling models for a diversity of both conifer and deciduous tree species (Baumgarten et al. 2021; Hänninen 2016; Sarvas 1974; Wang et al. 2024), fruit crops (Guak and Neilsen 2013; Mahmood et al. 2000), and vine cultivars (North et al. 2024). To our knowledge, few studies have compared time to budbreak (TBB) and endodormancy break under both natural and artificial chilling conditions in cold climates, where freezing temperatures are common in the winter.

Another confounding aspect of chilling units' computation that is often not considered is the influence of frost hardiness on dormancy depth. Most chilling‐forcing experiments evaluate TBB without accounting for the plant frost hardiness at the time of transfer to forcing conditions. However, recent studies have highlighted that samples under higher frost hardiness take more time to deacclimate and perform budbreak (Kovaleski et al. 2018; North and Kovaleski 2024). For example, a study by Kovaleski (2022) found that frost hardiness at the time of transfer to forcing conditions explained differences in TBB in 15 woody perennial species spanning the seed plant phylogeny. Controlled conditions at stable chilling temperatures can decrease plant frost hardiness compared to fluctuating natural conditions, leading to faster budbreak under forcing conditions (North and Kovaleski 2024). These studies highlight the importance of investigating the link between frost hardiness and TBB in chilling‐forcing experiments.

In this study, we performed chilling‐forcing experiments in saplings belonging to seven sugar maple ( *Acer saccharum* Marsh.) provenances in Eastern Canada. Our aim was to quantify the TBB after exposure to artificial and natural chilling treatments, testing for different chilling models and the influence of frost hardiness on budbreak. We predicted that: (1) endodormancy break would be easier to detect in artificial chilling treatments, where the confounding effect of frost hardiness would be limited; (2) a chilling model considering freezing temperatures would be more effective for a cold temperate species experiencing temperatures below 0°C for several months; and (3) sugar maple samples with higher frost hardiness would take more TBB.

## 2 Materials and Methods

### 2.1 Plant Material

**FIGURE 1:**
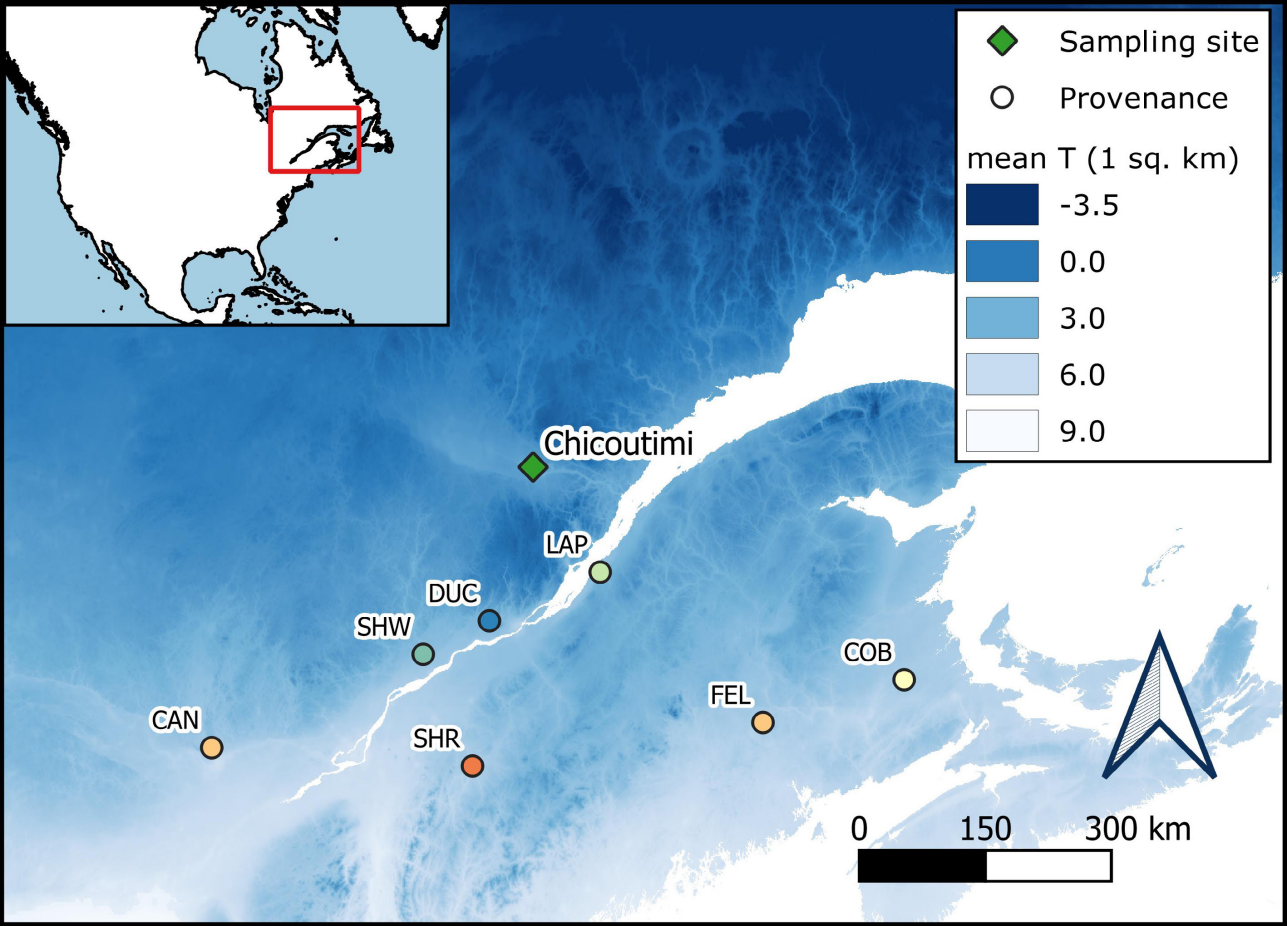
Sampling site (green diamond) and provenances (points) for sugar maple seedlings used in this study. Shades of blue indicate the mean annual temperature (°C) per pixel (1 km^2^).

This study used sugar maple ( *A. saccharum* Marsh.) seedlings of seven provenances produced by a forest nursery in Berthierville, QC, Canada (Table 1, Figure 1). The term “provenance” is used to indicate the geographic origin of plant material. Provenances Duchesnay, Coy Brook, and First Eel Lake (DUC, COB, and FEL, respectively) were collected on single mother trees by the National Tree Seed Center (Natural Resources Canada, Fredericton, Canada). Seeds for the Shawinigan, Lapocatière, Cantley, and Sherbrooke provenances (abbreviated as SHW, LAP, CAN, and SHR, respectively) were collected at stand level by the Ministère des Ressources Naturelles et des Forêts du Québec, Canada. All sites of seed collection are natural (i.e., no artificial selection or tree breeding) and are thus considered representative of the provenance area.

**TABLE 1:** Characteristics of the seven sugar maple provenances examined in this study. Climate data is relative to the 1970–2000 period (Source: WorldClim).

Provenance	ID	Elevation (m a.s.l.)	Annual temperature (°C)	Average minimum temperature of the coldest month (°C)	Annual precipitation (mm)
Duchesnay	DUC	250	3.4	−18.9	1364
Shawinigan	SHW	124	3.95	−18.8	1063
La Pocatière	LAP	22	4.21	−16.3	939
Coy Brook	COB	89	4.83	−15.2	1119
Cantley	CAN	154	4.88	−17.2	994
First Eel Lake	FEL	177	4.88	−16.7	1100
Sherbrooke	SHR	301	5.38	−15.9	1077

Seedlings germinated in 2020 at the forest nursery and grew in transparent plastic tunnels until they reached approximately 25 cm in height, at which point they were transferred outdoors and kept under a shading net. After the end of the growing season, seedlings were transferred into a cold room at −3°C for the rest of the winter. In May 2021, the seedlings were transplanted into trays containing 15 cavities of 320 cm^3^ and placed outdoors in Chicoutimi, Canada (named sampling site, Figure 1). Chicoutimi is located at the northern limit of the sugar maple range, with an average annual temperature of 2.8°C and an average minimum temperature of −22.1°C in the coldest month (Environment Canada 2023; Godman et al. 1990). Seedlings spent the following growing seasons outdoors under a shading net until the experiments, which started in late 2022.

### 2.2 Experimental Design

**FIGURE 2:**
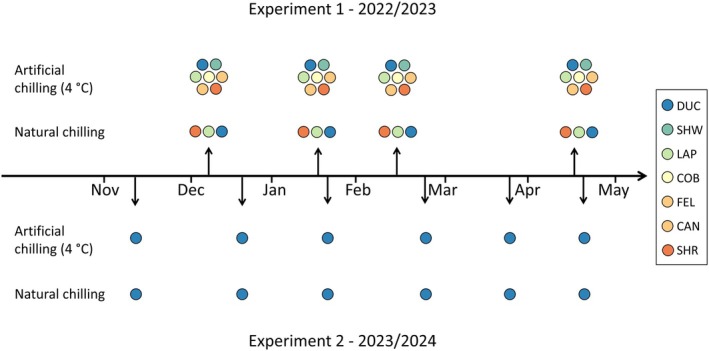
Experimental design adopted in this study and divided into two experiments during winter 2022–2023 (experiment 1, top panel) and winter 2023–2024 (experiment 2, lower panel). Arrows indicate dates of transfer to forcing treatment. Each point symbol indicates a different sugar maple provenance. Note that only one provenance (DUC) was analyzed in experiment 2.

We performed two experiments to assess chilling requirements in sugar maple seedlings, which took place in the winter 2022/2023 (hereafter experiment 1) and 2023/2024 (experiment 2) (Figure 2). Following leaf fall (12 December in experiment 1, 9 November in experiment 2), seedlings were either placed in growth chambers (Conviron models CMP6050 and CMP6060) for artificial chilling treatment (4°C, 8 h photoperiod) or left outdoors in natural conditions (which represents a colder treatment of chilling, with temperatures in the study area remaining < 0°C for most of the winter). Over the winter, saplings were sampled to obtain twig sections, named cuttings, of at least 5 cm, including the apical bud. Samplings were destructive, that is, different individuals were sampled on different dates (no repeated measurements). Cuttings were placed in trays with the bottom tips immersed in water and transferred to growth chambers at forcing treatment for budbreak observations.

Experiment 1 used all seven provenances of sugar maple. DUC, LAP, and SHR provenances were placed in both artificial and natural chilling treatments, while the other four provenances were only artificially chilled due to seedling availability. Over the course of chilling, we performed four transfers to forcing treatments (20°C, 16 h photoperiod, 75% RH) (Figure 2) at different times. We chose 16 h daylight for forcing conditions because it is the local daylength during the growing season, and it removes any potential limitations to growth from daylength. Transfer dates during winter 2022/2023 were 12 December (DOY 346), 16 January (DOY 16), 13 February (DOY 44), and 17 April (DOY 107). On each transfer date, 10 seedlings from each provenance and each chilling treatment were sampled for observations.

During experiment 2, we only used seedlings from the DUC provenance. We performed six transfers to forcing treatment (18°C, 16 h photoperiod, 75% RH) during the 2023/2024 winter. Transfer dates were: 9 November (DOY 313), 18 December (DOY 352), 22 January (DOY 22), 28 February (DOY 59), 26 March (DOY 86), and 22 April (DOY 113). On each transfer date, 15 seedlings per chilling treatment were sampled for budbreak and frost hardiness assessment. A lower forcing temperature was used in experiment 2 due to desiccation observed during experiment 1. It should be noted that DUC is one of the provenances collected at the tree level, which can be a limiting factor for the genetic diversity between samples. We chose this specific provenance because of the higher number of available samples.

### 2.3 Budbreak Observation

Once transferred to forcing treatment, buds were observed twice a week to assess budbreak. We used a phenological scale adapted from Skinner and Parker (1994) shown here with the corresponding BBCH stage (Meier 2001): (0) dormant bud, no sign of swelling (BBCH stage 00); (1) bud elongation, yellowish color visible between the scales (BBCH 01); (2) budbreak, with leaves visible between the scales (BBCH 09); (3) leaf emergence from the bud, leaves still not fully expanded (BBCH 10); and (4) complete leaf expansion (BBCH 11). TBB was defined as the number of days necessary to reach stage 2 after transferring the cuttings to forcing treatment.

### 2.4 Frost Hardiness Measurements

Frost hardiness was measured for buds and shoots using the REL (Relative Electrolyte Leakage) technique (Repo and Lappi 1989). On each transfer date, seven seedlings per chilling treatment (artificial vs. natural) were sampled for frost hardiness measurement. Three cuttings at least 5 cm long were obtained from each seedling, wrapped in tin foil, and randomly distributed between seven thermal containers. Thermal containers were then placed in a controlled‐temperature freezer (CryoMed controlled rate freezer, Thermo Fisher Scientific) and exposed to freezing temperatures. On each transfer date, we tested seven target temperatures between +5°C and −80°C. The cooling rate was set at 6°C h^−1^, the slowest reached by the controlled‐temperature freezer. After exposure to freezing temperatures, samples were placed in vials with 10 mL demineralized water. Conductivity in the vials was measured a first time after the target temperature treatment (C1) and a second time after an autoclave treatment to damage all the cells (C2, 120°C for 30 min). The ratio between C1 and C2, named REL, is a proxy for cellular damage caused by frost. We modelled REL values by temperature treatments as a logistic curve in order to identify LT_50_, that is the temperature inflicting 50% of cellular damage (Repo and Lappi 1989). More detailed information on the REL technique, including plots of the data points and fitted logistic curves, is provided in Supporting Information (Figure S1).

### 2.5 Weather and Climate Data

We obtained long‐term (1980–2010) climate averages to describe the sampling site (Chicoutimi) and the seven provenances with WorldClim (Fick and Hijmans 2017). For the winters of experiments 1 and 2, temperature data were measured hourly with an on‐site temperature weather station (Priva North America Inc., Vineland Station, Ontario, Canada). Missing data were replaced by data from the nearest available weather station, Bagotville (9 km from Chicoutimi, Environment Canada 2023).

We calculated hourly chilling units accumulation using four chilling metrics starting from 1 September: Chilling Hours as suggested by Weinberger (1950), Freezing Hours calculated using the same formula as Chilling Hours, but including temperatures below 0°C (higher and lower temperature limits at 7.2°C and −10°C, respectively), Chill Units according to the Utah Model (Richardson et al. 1974), and Chill Portions according to the Dynamic Model (Fishman et al. 1987). We used the *chilling_hourtable* function in the R package *chillR* (Luedeling et al. 2023) to calculate Chilling Hours, Chill Units, and Chill Portions. We applied a custom function based on Weinberger (1950) to calculate Freezing Hours. We chose −10°C as the lower chilling threshold as it was recently identified as an effective temperature for dormancy release (Wang et al. 2024).

### 2.6 Statistical Analysis

We tested the normality of budbreak dates for each treatment using the Shapiro–Wilks test and the homogeneity of variance using Bartlett's test. We used Pearson's Chi‐squared test to compare the proportion of buds performing budbreak in artificial and natural chilling treatments. The *p*‐value of reference for statistical significance was 0.05.

We used ANCOVA to test for significant effects of several variables on TBB. We compared several ANCOVA models, each using a different quantitative covariate (either time of transfer since start of the experiment in days, Chilling Hours, Chill Units, Chill Portions, or Freezing Hours) with chilling treatment (artificial vs. natural) as a categorical covariate. For experiment 1, provenance was also included as a categorical covariate. We used delta‐AIC to compare models with different quantitative variables to identify the best one (Akaike Information Criterion, Akaike 1974). Model goodness‐of‐fit was evaluated by adjusted *R* ^2^ values, distribution of standardized residuals, and visual assessment of diagnostics plots.

We fitted an exponential curve to test for the relationship between TBB and frost hardiness:

$$\mathrm{TBB}=a*e^{\left(b*{\mathrm{LT}}_{50}\right)}$$

where TBB is the time to budbreak (days) after transfer to forcing conditions, LT_50_ is frost hardiness at the time of transfer, and *a* and *b* are the terms of the function.

In order to identify the date of endodormancy break, that is, the moment in which further chilling accumulation does not reduce TBB, we applied segmented regression with TBB as the response variable and the days since the start of the experiment as the explanatory variable. We used the *segmented* package in R (Muggeo 2008). All statistical analyses were performed in R version 4.3.1 (R Development Core Team, 2023).

## 3 Results

**FIGURE 3:**
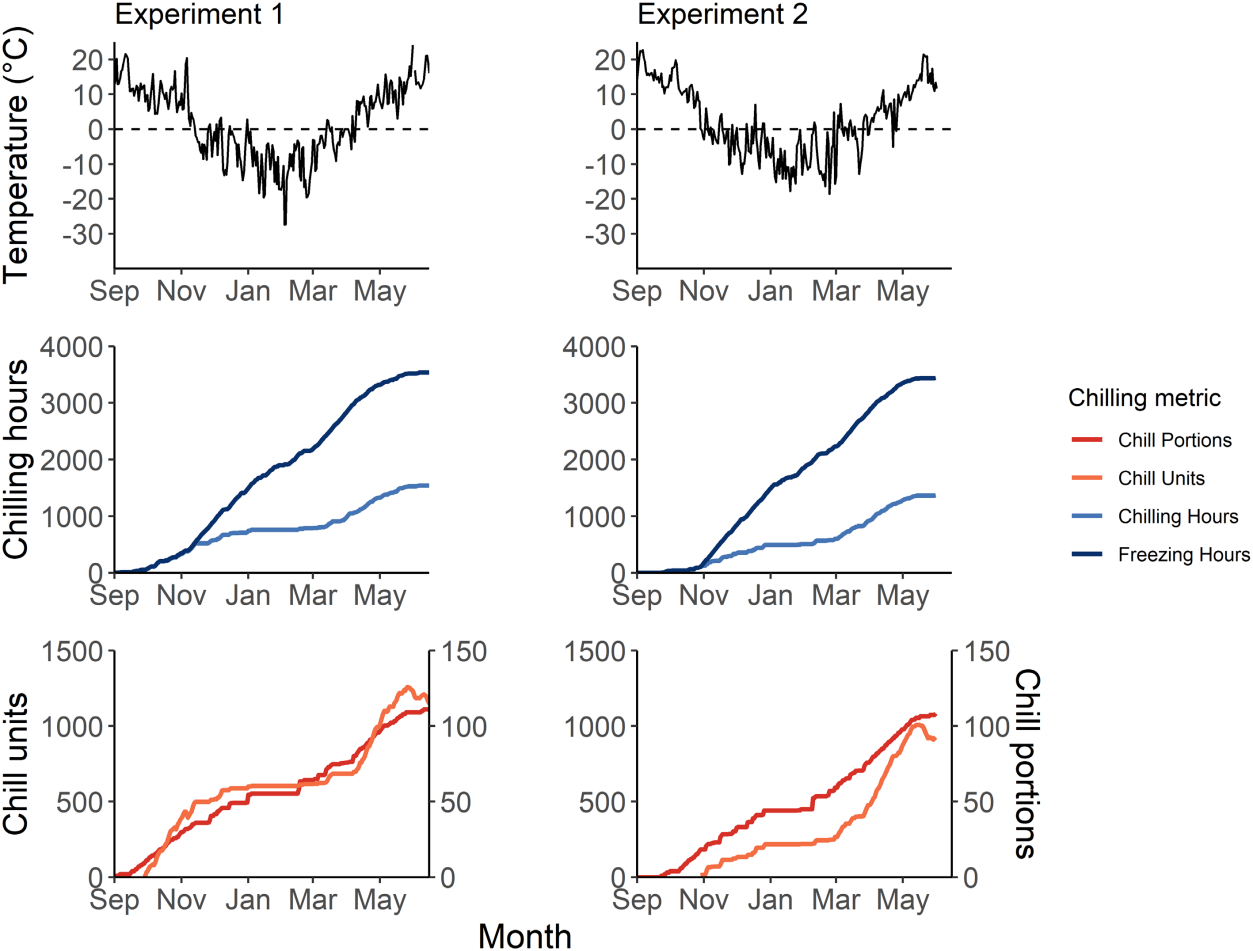
Daily air temperatures (top), accumulation of chilling metrics based on Weinberger 1950 (middle) and accumulation of chilling metrics from the Dynamic and Utah models (bottom). Freezing Hours is the only chilling metric accounting for the chilling effect of freezing temperatures. Chill Portions, Chill Units, and Chilling Hours are classic chilling metrics that only consider above‐freezing temperatures.

During experiment 1, temperatures below 0°C began occurring under natural conditions on 14 November. Mean daily temperatures remained below 0 for most of the winter, from 31 December 2022 until 14 March 2023. As a result, the accumulation of classic chilling metrics (i.e., not accounting for freezing temperatures) during this period was very low, amounting to 101 for Chilling Hours, 18 for Chill Portions, and 50 for Chill Units. By contrast, the Freezing Hours model, accounting for freezing temperatures, accumulated a total of 997 h over the same period. The weather during experiment 2 showed a similar pattern, with temperatures remaining mostly below 0°C between 18 December 2023 and 28 February 2024, resulting in low chilling accumulation for classic chilling models, while the Freezing Hours model kept increasing (Figure 3).

**FIGURE 4:**
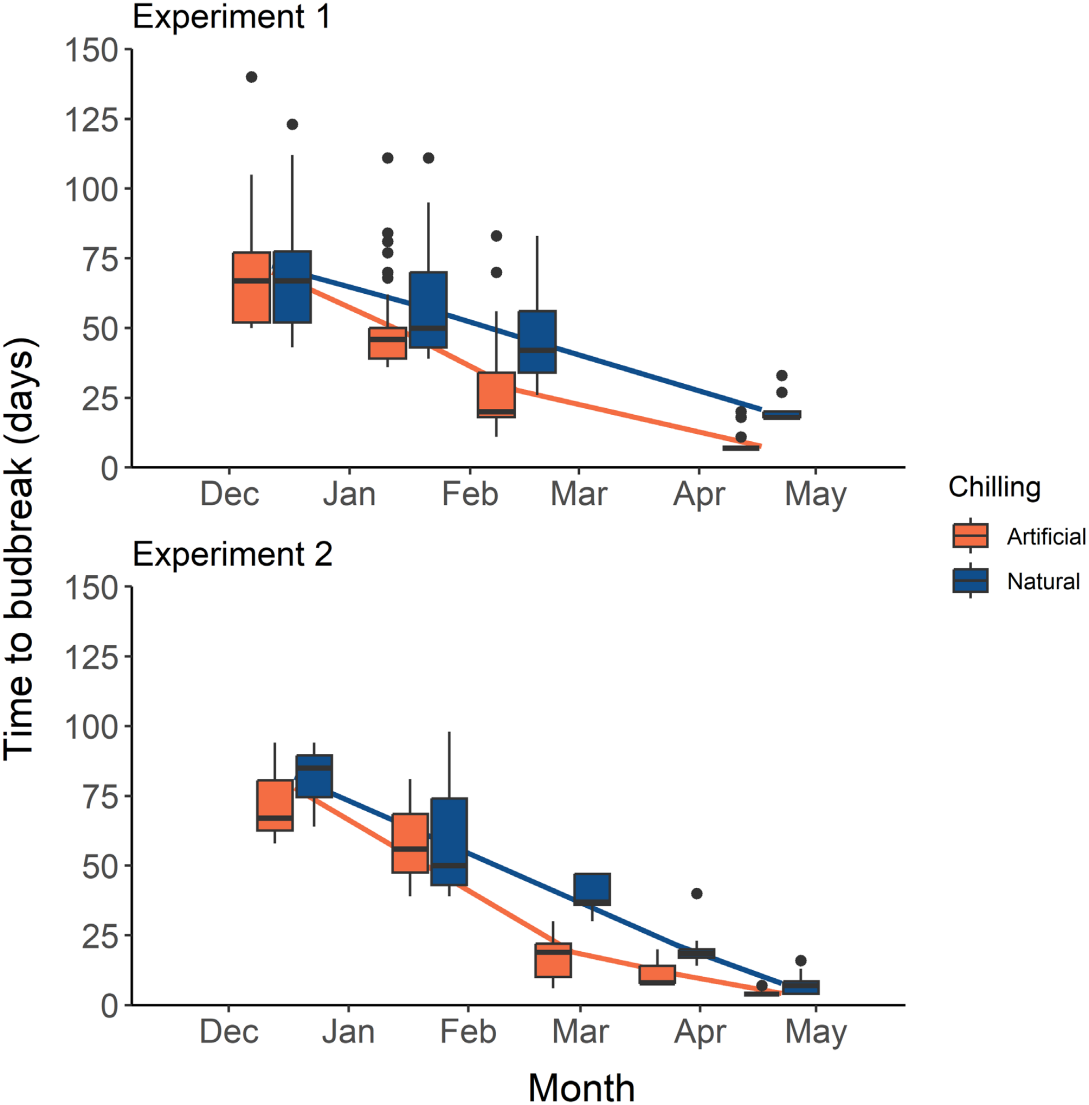
Boxplots of time to budbreak (TBB) in sugar maple seedlings transferred to warm treatments during experiments 1 (winter 2022–2023) and 2 (winter 2023–2024). Different colors indicate whether the samples were exposed to artificial (orange) or natural (dark blue) chilling treatments prior to sampling. Boxplot lines indicate the median and quartiles, while points indicate outlier values. Lines indicate the segmented regression model used to identify endodormancy break. Corresponding statistical values for the segmented regression are shown in Table 4.

Time to budbreak decreased over time in both experiments (Figure 4). During experiment 1, TBB decreased from 70 ± 20 days on the first transfer date (12 December 2022) to 12 ± 7 days on the last transfer date (17 April 2023). During experiment 2, TBB decreased from 77 ± 16 days to 6 ± 3 days. During both experiments, some samples attained stage 1 of the budbreak process (bud elongation) under artificial chilling treatments in April. In the ANCOVA models, Delta‐AIC and *R* ^2^ indicated that time and freezing hours were the best quantitative covariates for TBB, while classic chilling metrics not accounting for temperatures below 0°C had lower performance in both experiments (Table 2). In the best ANCOVA model for experiment 1, Freezing Hours, chilling treatment, and their interaction had a significant effect on TBB, while provenance was not significant (Table 2). In experiment 2, the best ANCOVA indicated a significant effect of time and chilling treatment, but not their interaction (Table 2). In both years, samples in artificial treatments showed a faster decrease of TBB over time (Figure 4).

**TABLE 2:** Results of ANCOVA models testing the effect of quantitative covariates, chilling treatment (artificial vs. natural), and provenance (only in experiment 1) on the time to budbreak (TBB) under forcing treatment.

	Time	Chill units	Chill portions	Chilling hours	Freezing hours
Experiment 1					
*R* ^2^	0.7	0.69	0.69	0.69	0.71
*F*	74.57***	68.63***	71.23***	69.99***	75.7***
AIC	2272.6	2281.7	2285.2	2289	2269.73
Quantitative variable	472.7***	138.85***	282.66***	175.79***	380.747***
Chilling treatment	8.19**	48.16***	5.2*	22.77***	5.62*
Provenance	1.89	1.74	1.8	1.78	1.9
Quantitative × chilling	3.36	90.61***	19.64***	70.96***	5.42*
Experiment 2					
*R* ^2^	0.82	0.7	0.79	0.76	0.82
*F*	126.2***	64.14***	100.5***	84.13***	125.3***
AIC	612.6	627.5	638.6	654.7	619.6
Quantitative variable	331.41***	130.44***	265.13***	188.5***	334.55***
Chilling treatment	5.37*	3.71	0.98	0.045	8.95**
Quantitative × chilling	1.43	45.71***	17.83***	56.96***	20.54***

*Note:* Quantitative variables are either Time (days since the start of the experiment) or a chilling accumulation metric (Chill Units, Chill Portions, Chilling Hours, or Freezing Hours). For the whole model, the adjusted *R* ^2^ for goodness of fit and *F* values and significance levels are shown. For the variables, *F* values and significance levels are shown. One, two, and three asterisks correspond to *p* < 0.05, *p* < 0.01, and *p* < 0.001, respectively. Degrees of freedom for the numerator/denominator are 9/270 for experiment 1 and 3/76 for experiment 2.

During both experiments, the budbreak percentage of samples increased with the duration of chilling (Figure S2). The percentage of successful budbreak varied from a minimum of 52% (12 December 2022, natural chilling) to a maximum of 100% (17 April 2023, artificial chilling) in experiment 1, and from a minimum of 6% (9 November 2023, natural chilling) to a maximum of 100% (22 April 2024, artificial chilling) in experiment 2. Samples in artificial chilling had higher budbreak percentages than samples in natural chilling in most cases. During experiment 1, samples in the artificial chilling treatment had a higher percentage of budbreak than those in the natural chilling treatment on both 13 February (*χ* ^2^ = 6.4, *p* = 0.01) and 17 April (*χ* ^2^ = 14.2; *p* < 0.001). Conversely, during experiment 2, samples in natural chilling had a higher budbreak percentage than samples in artificial chilling on 28 February (*χ* ^2^ = 3.9; *p* = 0.049), with no significant differences on other dates.

**FIGURE 5:**
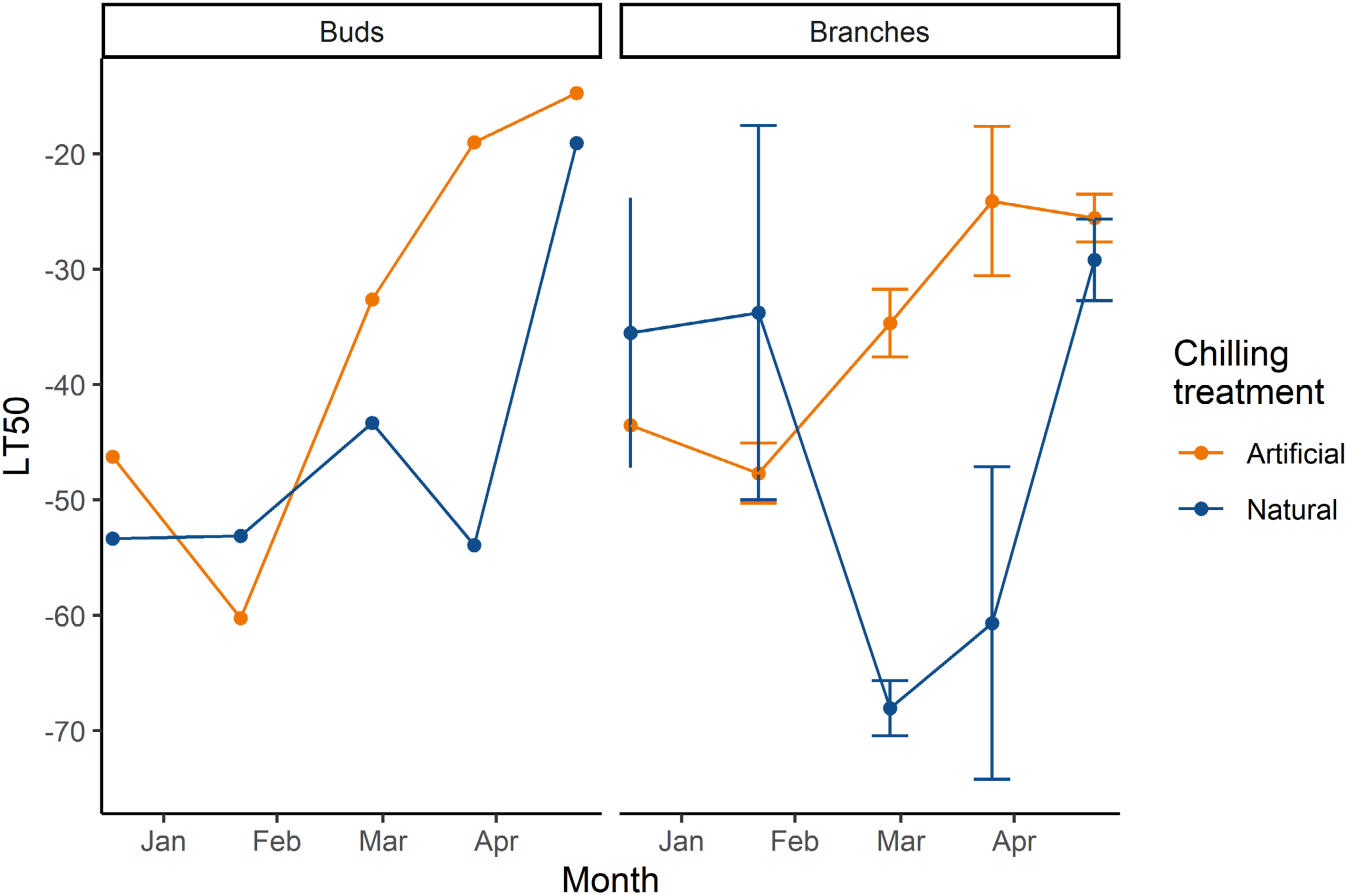
LT_50_ values in sugar maple buds and branches under two different artificial (orange) and natural (dark blue) chilling treatments during experiment 2 (winter 2023–2024). Dots and error bars indicate average and standard deviation, respectively, for LT_50_ values calculated for each sampling date using the REL method. Values for buds were obtained from one estimate, because of the limited amount of plant material (see Supporting Information), and therefore are shown without standard deviation.

**FIGURE 6:**
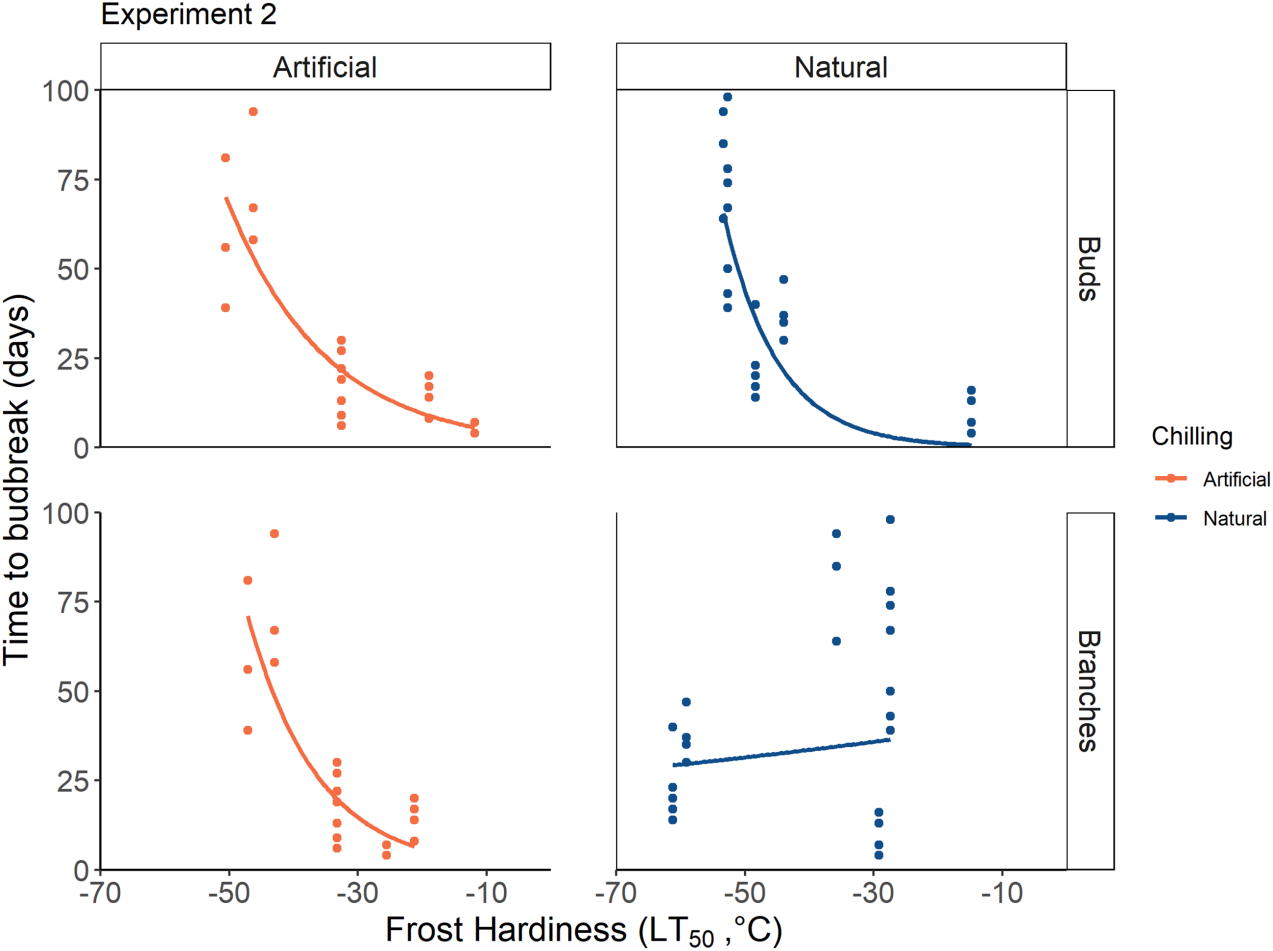
Relationship between time to budbreak (TBB) under forcing treatment and frost hardiness for sugar maple under artificial (orange) and natural (dark blue) chilling treatments. Points show LT_50_ (°C) estimation using the REL method. Lines refer to the exponential curve fit of the data. “Ps.R2” stands for Pseudo *R* ^2^. Corresponding statistical values for the curve fitting are shown in Table 3.

During experiment 2, frost hardiness, based on LT_50_, varied between −53°C and −12°C in buds, measured on 18 December 2023 and 22 April 2024, respectively (Figure 5). LT_50_ in branches was slightly lower and varied between −61°C and −15°C, measured on 26 March and 27 May 2024, respectively (Figure 5). LT_50_ in buds reached its lowest values between December and mid‐January and consistently increased afterwards in both natural and artificial chilling treatments. LT_50_ in branches in artificial chilling followed a similar pattern to buds, increasing from January onwards. Conversely, LT_50_ in branches under natural treatment showed a less linear pattern, with a marked decrease between 28 February and 26 March. A negative exponential relationship was observed between bud LT_50_ and TBB, where higher bud frost hardiness (i.e., lower LT_50_ values) corresponded to longer TBB, for both natural and artificial chilling (Figure 6). A similar relationship was found for branch LT50 and TBB under artificial conditions, but not under natural chilling (Figure 6, Table 3).

**TABLE 3:** Results of exponential curve fitting for the relationship between time to budbreak (TBB) and frost hardiness.

Organ	Chilling	RSS	RMSE	Pseudo *R* ^2^	Term	Estimate	SE	*p*
Buds	Artificial	4232	10.04	0.79	a	2.578	0.80	0.003**
					b	−0.065	0.01	< 0.001***
	Natural	10,848	16.27	0.63	a	0.111	0.15	0.462
					b	−0.120	0.03	< 0.001***
Branches	Artificial	5388	11.33	0.73	a	0.927	0.41	0.029*
					b	−0.092	0.01	< 0.001***
	Natural	27,331	25.82	0.02	a	43.543	15.38	0.007**
					b	0.007	0.01	0.445

*Note:* Residual sum of squares (RSS), root mean square error (RMSE), and Pseudo *R* ^2^ values are shown for the overall fit, as well as estimate, standard error, and *p*‐value estimation for the model terms. One, two, and three asterisks correspond to *p* < 0.05, *p* < 0.01, and *p* < 0.001, respectively. Degrees of freedom for the numerator/denominator are 1/39 for natural chilling and 1/40 for artificial chilling. Fitted exponential curves are shown in Figure 6.

Segmented regression suggested that sugar maple broke endodormancy (minimized TBB) on 13 February in experiment 1 (Table 4). The same date was estimated for both artificial and natural chilling treatments. For experiment 2, the endodormancy break date was estimated on 2 March and 26 March in artificial and natural chilling, respectively. For both experiments, the estimation for natural chilling had a much higher standard deviation due to the linear pattern of TBB decrease, making the estimation unreliable (Figure 4). The Dynamic model (Fishman et al. 1987) produced the smallest difference between dates of endodormancy across years for artificial chilling. Coefficients of variation of the date of endodormancy break across years ranged between 0.02 and 0.04 for all chilling metrics.

**TABLE 4:** Timings of endodormancy break identified by segmented regression. Standard deviation of the estimated date of dormancy break is shown.

	Experiment 1		Experiment 2	
	Artificial	Natural	Artificial	Natural
Dormancy break	2023‐02‐13	2023‐02‐13	2024‐03‐02	2024‐03‐26
SD (days)	12	741	10	71
DF	211	88	38	37
Chilling hours	2715	762	2968	834
Chill portions	103.9	55.3	108.5	71.2
Chill units	2711.5	604.5	2830.5	405
Freezing hours	2734	2022	3669	2743

*Note:* Cumulative values for several chilling metrics are also reported for each dormancy break date: Chilling Hours as suggested by Weinberger (1950), Chill Portions according to the Dynamic Model (Erez et al. 1990), Chill Units according to the Utah Model (Richardson et al. 1974), and Freezing Hours according to the modified Weinberger (1950) model including temperatures down to −10°C. Segmented regression lines are shown in Figure 4. DF indicates degrees of freedom.

## 4 Discussion

Time to budbreak under forcing conditions decreased with increasing exposure to chilling temperatures during the winter. The rate of decrease was faster under artificial stable chilling compared to natural chilling. A cumulative chilling metric accounting for mild temperatures below 0°C (Freezing Hours) was a better predictor than classic metrics (Chilling Hours, Chill Units, and Chill Portions, only considering temperatures above 0°C). This suggests that accounting for below‐freezing temperatures improves the measurement of chilling accumulation in cold temperate species such as sugar maple.

Endodormancy break was only reliably detectable in samples under artificial chilling conditions. We observed that the artificial chilling at 4°C induced earlier deacclimation, initiating ontogenetic development in the bud in the first half of April (before transfer to forcing conditions). Conversely, frost hardiness measurements indicated that samples in natural conditions retained higher frost hardiness levels until late spring. Samples were able to reach LT_50_ below −50°C until the end of March, a temperature well below the average minimum winter temperatures common in the study area (−33.4°C ± 2.7°C for the 1990–2020 period, Environment Canada 2023). Higher frost hardiness correlated with longer TBB, possibly suggesting a confounding effect of frost hardiness on TBB. These results highlight the importance of measuring frost hardiness when performing chilling‐forcing experiments, especially in cold climates where frost damage is a risk until late in the growing season.

### 4.1 Chilling Temperatures

The timing of transfer from chilling to forcing conditions and Freezing Hours were the best explanatory variables for the time necessary to budbreak (Table 2). Interestingly, Freezing Hours (i.e., the chilling model including freezing temperatures down to −10°C) outperformed classic chilling metrics (i.e., Chilling Hours, Chill Units, and Chill Portions). This suggests that accounting for temperatures below 0°C in chilling accumulation improved the prediction of TBB, confirming our hypothesis. It should be noted that, in our study, classic chilling units still had reasonably high *R* ^2^ values, indicating a good overall performance. However, Chilling Hours and Chill Units, in particular, seem to underestimate chilling accumulation in natural conditions (Figure S3).

This could be explained by the rigid winter temperatures, remaining below 0°C during several months. As a result, classic chilling models (Chilling Hours, Chill Units, and Chill Portions) calculated virtually no chilling accumulation over much of the winter (Figure 3). Indeed, classic chilling models were first developed to predict fruit tree phenology in temperate climates (Fishman et al. 1987; Richardson et al. 1974; Weinberger 1950), with no consideration of chilling accumulation below 0°C. This assumption (i.e., that chilling accumulation happens only above 0°C) therefore does not hold in temperate and cold boreal climates and can lead to an underestimation of chilling requirements, negatively affecting endodormancy break calculation and thus predictions under future climate conditions. Other studies have shown the importance of including freezing temperatures in chilling accumulation (Baumgarten et al. 2021; Guak and Neilsen 2013; Hänninen 1990; Mahmood et al. 2000; North et al. 2024). A recent study evaluated the effectiveness of different chilling models using vast phenological databases and found that models accounting for freezing temperatures performed better than those limited to temperatures above freezing (Wang et al. 2020).

The conviction that there is no chilling accumulation below 0°C probably stems from the assumption that molecular processes, involving the mechanisms of endodormancy release, cannot take place in the frozen state. However, trees can avoid ice formation by limiting ice nucleation in the tissues through supercooling (Bozonnet et al. 2024; Neuner et al. 1999; Pramsohler et al. 2012), which could allow the molecular processes involved in endodormancy break to function below 0°C. The main problem in explaining the effective temperature range that releases endodormancy comes from the fact that the underlying physiological regulation of endodormancy break is still largely unknown (Cooke et al. 2012; Hänninen et al. 2019). Until we have a more mechanistic understanding of the physiological and molecular processes underlying the chilling requirement and endodormancy break, temperature ranges for chilling accumulation models remain based on experimental evidence and assumptions.

Our results show that between 2715 and 3075 h at 4°C are necessary to break endodormancy and minimize TBB. While these results do not reflect natural conditions, they are still relevant for both comparison with other studies of chilling at mild temperatures and to clarify endodormancy break in sugar maple under artificial conditions. In a chilling‐forcing experiment on several provenances of sugar maple, Kriebel and Wang (1962) found that the greatest reduction in TBB took place between 1235 and 1637 chilling hours below 7.2°C. The difference with our results could be explained by the different provenances used in the studies. We selected maples from the northern part of the species' range, while Kriebel and Wang (1962) used samples from a wider gradient including warmer regions of the native range. Kriebel and Wang (1962) did find that northern provenances have higher chilling requirements, which would explain the high values observed in our study.

Little variation in chilling requirements and budbreak was found between provenances. This lack of differences could be explained by the limited geographic gradient considered in our study. Our provenances were all located in eastern Canada, an area corresponding to the northern portion of sugar maple's range (Godman et al. 1990). Studies considering provenances over a wider geographic gradient may find more pronounced differences. For example, Kriebel (1957) compared budbreak in sugar maple provenances from a wider area ranging from Tennessee (US) to Québec (CA) and found three main ecotypes, namely “southern”, “central” and “northern”. It is likely that the samples used in our study belong to the northern ecotype, closer to the range edge, and have a lower intraspecific difference (Perry and Knowles 1989; Young et al. 1993).

The lack of differences in budbreak timings suggests that provenance selection in forestry has a limited role to play in reducing the risk of late frost damage. Other tools may be more effective, such as choosing a plantation site sheltered from extreme frosts or adopting silvicultural techniques such as a shelterwood system to create a more favorable microclimate (Charrier et al. 2015; Dumais et al. 2024). This is particularly relevant for sugar maple, as the species is shade‐tolerant and can grow well in a sheltered site where an upper canopy is present (Boulet 2013; Godman et al. 1990).

### 4.2 Budbreak and Frost Hardiness

The time to reach budbreak decreased as transfers to forcing conditions became closer to spring (usually late April/early May in the study area). As chilling exposure accumulates, trees enter ecodormancy and become more sensitive to external conditions, decreasing the time necessary to budbreak (Charrier et al. 2011; Chuine et al. 2016; Lang et al. 1987). TBB decreased faster under artificial chilling treatment (4°C). In most cases, artificial chilling also led to higher percentages of budbreak. At first glance, this could suggest that mild and stable chilling conditions (at 4°C) were more effective than fluctuating natural temperatures in fulfilling chilling requirements, as suggested by the shape of the relation between temperature and chilling hours or chill units (Figure S3). However, we argue that bringing frost hardiness into the picture may offer an alternative explanation, as mild artificial chilling may have both fulfilled the chilling requirement and contributed to forcing, initiating ontogenetic development in the bud (Charrier et al. 2011). Conversely, low freezing temperatures in natural conditions could have fulfilled the chilling requirement but also induced higher levels of frost hardiness and prevented deacclimation until late in spring.

While temperatures of 4°C were cold enough to fulfill chilling requirements in sugar maple, artificial chilling promoted deacclimation to frost, which could potentially be detrimental for trees. In late March, bud LT_50_ under artificial and natural conditions was −18°C and −48°C, respectively, highlighting the earlier decrease of frost hardiness under artificial conditions. Temperatures lower than −18°C are not uncommon in the study area in April, and highlight the potential risk of frost damage following deacclimation under warming winter conditions. The earlier deacclimation under artificial chilling was also confirmed by visual observation of buds initiating the budbreak process inside the growth chambers at 4°C in April, before the transfer to forcing conditions. Other studies report that above‐freezing chilling temperatures can induce ontogenetic development once chilling requirements are fulfilled (Hänninen 1990). Kovaleski (2022) found similar results in red maple ( *Acer rubrum* L.), which lost around 0.2°C in LT_50_ per day at 4°C after fulfilling the chilling requirement. Both sugar maple and red maple are species with wide distribution, which spans northwards until the limit of the boreal forest, and these species may be particularly sensitive to mild temperatures above 0°C in the spring. Conversely, species accustomed to warmer climates may be less sensitive. Furthermore, Charrier et al. (2011) observed budbreak on European walnut ( *Juglans regia* L.) in samples kept at 5°C artificial chilling conditions, but at very low rates (i.e., budbreak date after more than 6 months).

Longer TBB was correlated with stronger frost hardiness in buds (i.e., lower LT_50_). This relationship was observed in both artificial and natural chilling, although it was stronger in natural conditions. In branches, we found a correlation between frost hardiness and TBB only under artificial chilling. Although correlation does not necessarily entail causation, frost hardiness could explain the observed differences in TBB between samples in artificial and natural chilling. Samples in the natural chilling treatment were exposed to freezing temperatures until late March, inducing lower LT_50_ and thus preventing quick deacclimation (Neuner et al. 1999; Vitasse et al. 2014) and potential cell damage. Kovaleski (2022) has recently demonstrated that both the level of frost hardiness at the time of transfer and the rate of deacclimation can explain the time required to reach budbreak in several tree species, including sugar maple. This correlation between frost hardiness and budbreak may be caused by the physiological mechanisms involved in increasing frost hardiness. These changes involve the osmotic potential of the cells (Bozonnet et al. 2024; Charrier et al. 2013) and the hydraulic connection between stems and buds (ice barrier; Neuner et al. 2019; Villouta et al. 2022). These adjustments are not compatible with growth resumption, budburst, and leaf formation, which require highly hydrated tissues connected to the stem (Hänninen 2016; Xie et al. 2018). Plants with higher levels of cold hardiness need to adjust physiologically before budbreak, that is by rehydrating their tissues and restoring connectivity between bud and stem, resulting in a longer TBB after transfer to forcing conditions as observed by Kovaleski (2022).

The identification of an endodormancy break point was only reliable in samples under artificial conditions. Indeed, TBB in samples under natural chilling showed a linear decrease without a break point (Figure 4). By contrast, samples in the artificial chilling treatment started deacclimating earlier, leading to a faster decrease of TBB and allowing for easy identification of the endodormancy break point. One possible explanation is that freezing air temperatures in natural chilling could have kept the samples at higher frost hardiness levels, inducing a series of physiological adjustments (dehydrated cells, increased sugar content, frost barrier between bud and stem). This would cause a concurrent effect, where plants in natural chilling have longer TBB than plants in artificial chilling because of the need to revert the physiological changes required to increase frost hardiness, independently of the actual fulfillment of chilling requirements. However, an alternative explanation could also be that very low freezing temperatures (e.g., below −10°C) prevented or delayed the fulfillment of chilling requirements by stopping metabolic reactions in the cells.

## 5 Conclusion

We performed chilling‐forcing experiments on sugar maple saplings for two consecutive winters. We tested the effect of natural and artificial chilling treatments and measured frost hardiness on each transfer to forcing conditions. Fulfillment of chilling requirements was easier to detect in the artificial chilling treatment, while cold temperatures in natural conditions induced strong acclimation until spring and masked the effect of endodormancy break. As a result, the TBB decreased faster in artificial chilling conditions. Accounting for freezing temperatures in chilling accumulation worked better than classic models. Provenance did not have a significant effect on TBB.

This study provides quantitative measurements of the chilling requirements of sugar maple, an important factor to account for in a changing climate scenario. Moreover, our results join a growing body of literature highlighting the importance of both measuring frost hardiness and accounting for freezing temperatures in chilling accumulation when performing chilling–forcing experiments. Taking into account frost acclimation and deacclimation dynamics can greatly improve the understanding and interpretation of chilling–forcing experiment results in the future. Future studies employing chilling–forcing experiments should account for deacclimation dynamics by performing regular frost hardiness (such as LT_50_ tests) measurements after the transfer to forcing conditions. Similarly, repeated measurements of LT_50_ under different chilling conditions could help quantify the contribution of mild chilling temperatures to forcing and deacclimation. These aspects can help clarify the role of above‐ and below‐freezing temperatures in regulating frost hardiness and dormancy, advancing the knowledge on this crucial and yet still partially understood aspect of plant physiology.

## Author Contributions

Design of the research: C.M., G.C., A.P.K., A.D., S.R.; Performance of the research: C.M., G.C., A.P.K., P.R., A.D., S.R.; Data analysis, collection, or interpretation: C.M., G.C., A.P.K., A.D., S.R.; Writing the manuscript: C.M., G.C., A.P.K., P.R., A.D., S.R.

## Supporting information


**Figure S1:** Plots of REL values by experimental freezing temperature. REL analyses were carried out monthly on the date of transfer from chilling to forcing conditions. Black dots indicate REL values (mean value for each target temperature) measured during Experiment 2, blue lines indicate logistic curves fitted to calculate LT_50_.
**Figure S2:** Percentage of buds performing budbreak after transfer to forcing treatment and divided in two experiments during winter 2022–2023 (experiment 1, top panel) and winter 2023–2024 (experiment 2, lower panel). Bar colors indicate artificial (orange) and natural (dark blue) chilling treatment. For experiment 1, all provenances are grouped together because of a non‐significant effect according to ANCOVA.
**Figure S3:** Time to budbreak by the main chilling metrics used in this study, under both natural and artificial chilling conditions.

## Data Availability

Data is available on request from the corresponding author.
