# Supplementary material for: Is It Cold Enough? Effects of Artificial and Natural Chilling on Budbreak and Frost Hardiness in Acer saccharum (Marsh.)
Source: Physiol Plant. 2025 Oct 15;177(5):e70586. doi: 10.1111/ppl.70586 (PMC12528974; doi:10.1111/ppl.70586)
Supplement: Supplementary file 1 — Figure S1: Plots of REL values by experimental freezing temperature. REL analyses were carried out monthly on the date of transfer from chilling to forcing conditions. Black dots indicate REL values (mean value for each target temperature) measured during Experiment 2, blue lines indicate logistic curves fitted to calculate LT50. Figure S2: Percentage of buds performing budbreak after transfer to forcing treatment and divided in two experiments during winter 2022–2023 (experiment 1, top panel) and winter 2023–2024 (experiment 2, lower panel). Bar colors indicate artificial (orange) and natural (dark blue) chilling treatment. For experiment 1, all provenances are grouped together because of a non‐significant effect according to ANCOVA. Figure S3: Time to budbreak by the main chilling metrics used in this study, under both natural and artificial chilling conditions. [file PPL-177-e70586-s001.docx]

# Supporting Information

## REL method for experiment 2

On each sampling date, seven seedlings per provenance were collected in each site. Seedlings were separated into three samples, each at least 5 cm long, and distributed randomly between seven target temperatures for the frost treatment. In total, each target temperature had 3 samples per provenance, wrapped in tin foil and placed in a thermal container. Thermocouples (two per container) were used to measure sample temperature during the tests.

During each test, we exposed the samples to seven different treatment temperatures ranging from +5 to -80 °C. One thermal container was stored in a cold chamber at +5 °C (control treatment). The remaining six thermal containers were exposed to five different temperatures ranging from -7 °C to -80 °C in a controlled-temperature freezer (CryoMed controlled rate freezer, Thermo Fisher Scientific). The target temperatures changed during the sampling dates to better quantify the expected frost hardiness (i.e. we tested colder temperatures during winter). The temperature in the freezer was manually adjusted to attain a cooling rate of -6 °C h^-1^. Upon reaching one of the target temperatures, one random container was taken out of the freezer and stored in a cold chamber at +5 °C. All containers were then left at +5 °C overnight.

On the second day, the samples were prepared for conductivity measurements. Samples were separated in branches (cut in slices 0.5 mm thick) and buds (split in two along the longitudinal axis), then stored in vials with 10 ml of demineralized water. Because of the small number of buds available, buds within the same target temperature were placed in the same vial. Vials were left to agitate on a multi-platform orbital shaker (Thermo Fisher Scientific, Waltham, MA) at 5 °C overnight.

The third day, conductivity in each tube was measured as an indicator of electrolyte leakage from cells damaged by the frost (C_1_). Samples were then put in an autoclave at 120 °C, 1 bar, for 30 minutes. A second conductivity measurement was performed after the autoclave treatment, corresponding to the maximum cellular damage (C_2_). REL was then calculated as C1/C2, *i.e.* the ratio of electrolyte leakage caused by frost to the leakage caused by maximum damage. We calculated the relationship between REL and temperature using the logistic function (Repo & Lappi, 1989):

|  | $REL=\frac{a}{\left( 1+e^{b\left( c-T \right)} \right)}+d$ | ( 1) |
| --- | --- | --- |

where *T* is the test temperature, *d* is the higher asymptote, *a+d* is the lower asymptote and *b* is the slope at the inflection point *c*. Frost hardiness (LT_50_) was calculated for each provenance as the temperature at the inflection point *c*, i.e. the temperature causing 50% of cellular damage. Temperatures inducing 10% cellular damage (LT_10_) were also calculated from the logistic curve, to estimate a lower threshold for frost damage occurrence and for comparison with LT_50_. The logistic function was fitted with the nlsLM function of the minpack.lm package (Elzhov et al., 2022).


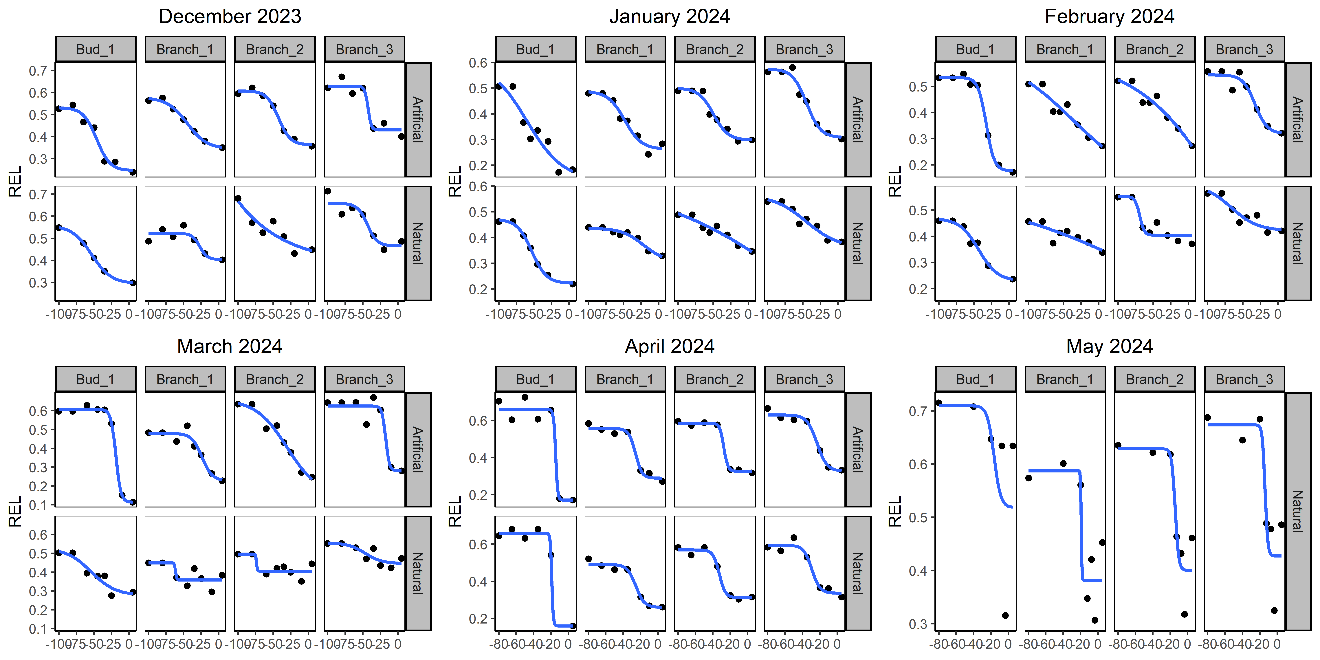


**Figure S1 :** Plots of REL values by experimental freezing temperature. REL analyses were carried out monthly on the date of transfer from chilling to forcing conditions. Black dots indicate REL values (mean value for each target temperature) measured during Experiment 2, blue lines indicate logistic curves fitted to calculate LT_50_.


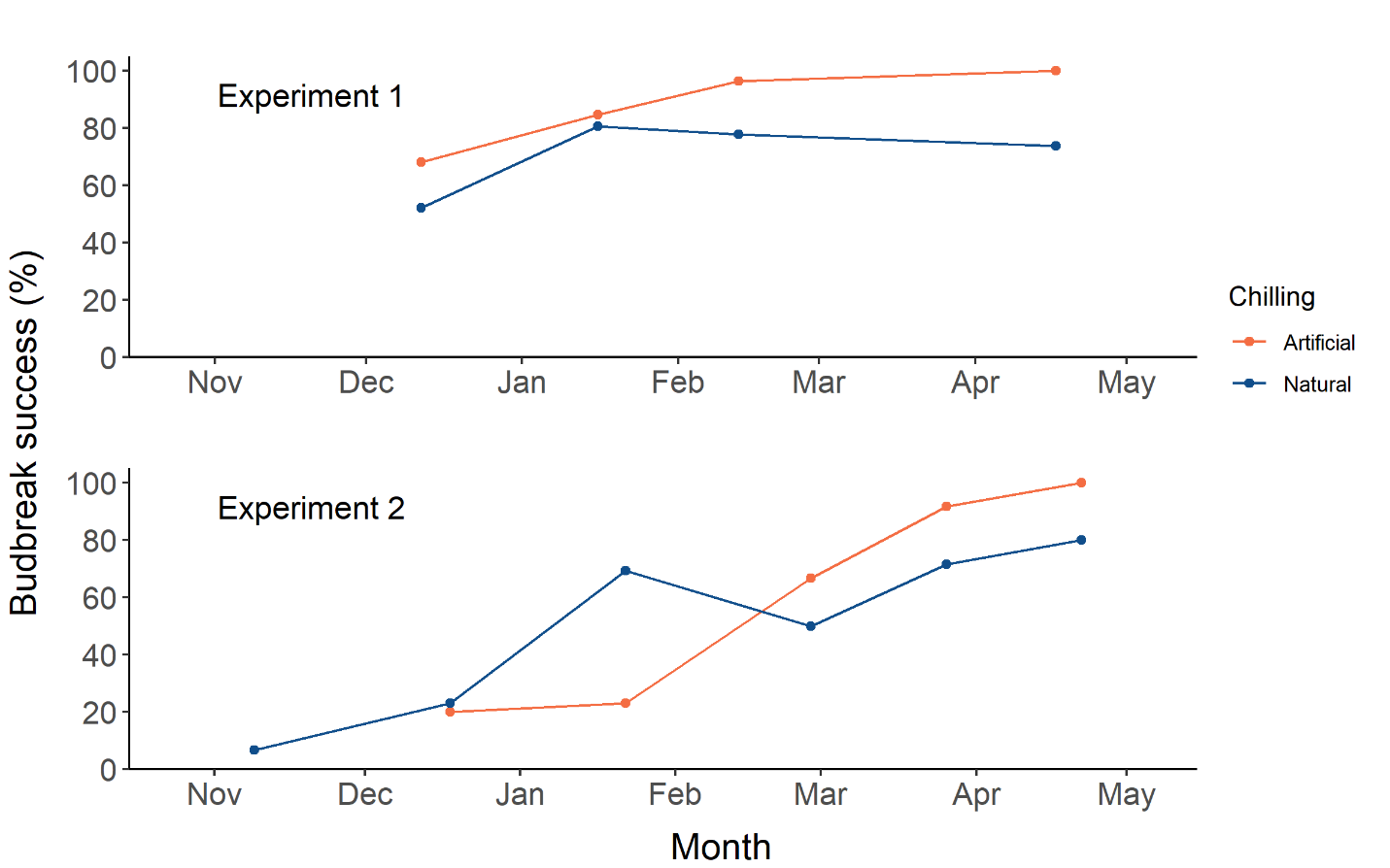


**Figure S2.** Percentage of buds performing budbreak after transfer to forcing treatment and divided in two experiments during winter 2022-2023 (experiment 1, top panel) and winter 2023-2024 (experiment 2, lower panel). Bar colors indicate artificial (orange) and natural (dark blue) chilling treatment. For experiment 1, all provenances are grouped together because of a non-significant effect according to ANCOVA.


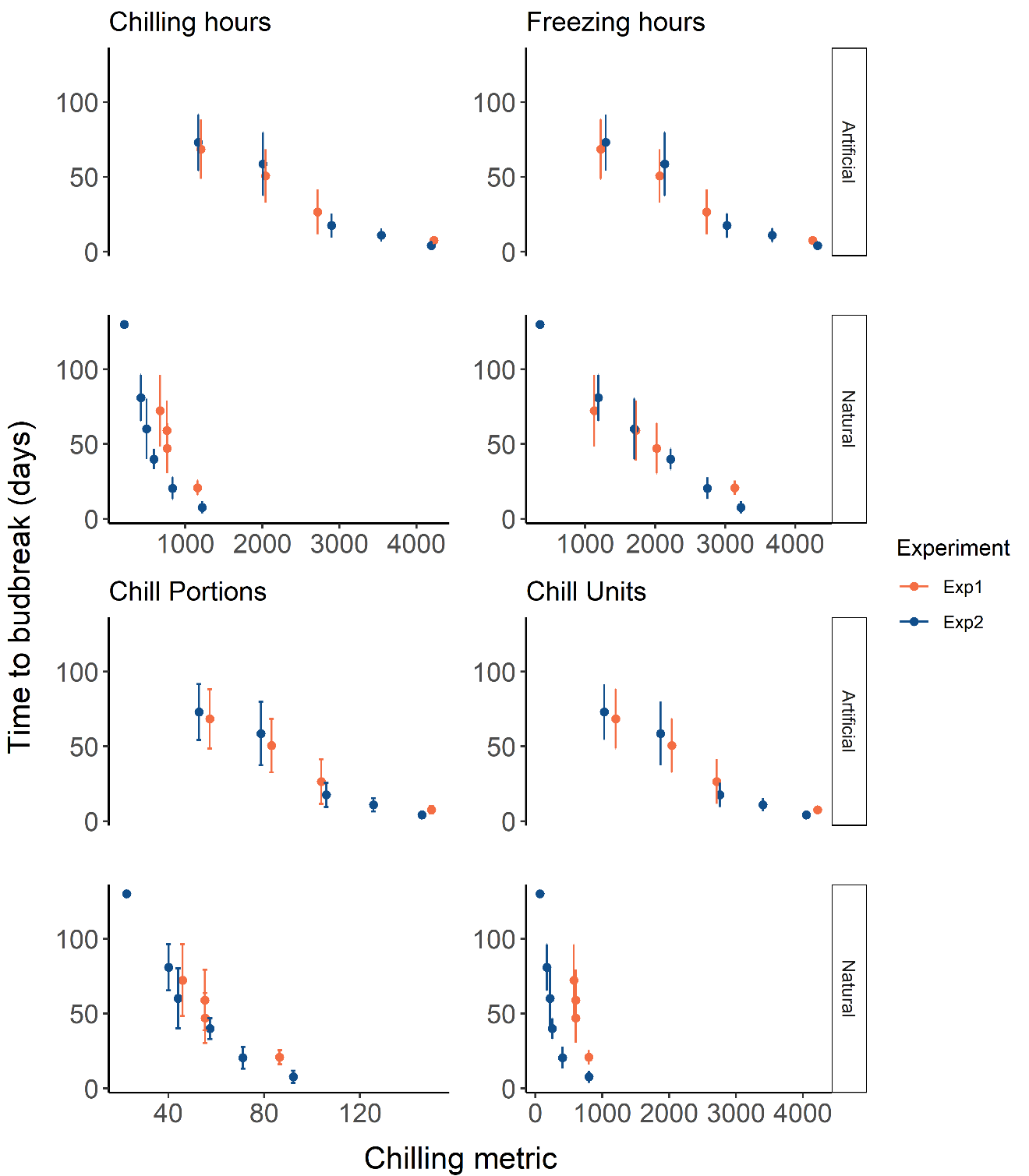


**Figure S3.** Time to budbreak by the main chilling metrics used in this study, under both natural and artificial chilling conditions.
